# *N*-propargylputrescine as a minimal-tag probe for imaging putrescine dynamics in cellular homeostasis and stress

**DOI:** 10.1016/j.isci.2026.117380

**Published:** 2026-09-03

**Authors:** Aleksandra Owczarek, Aleksandra Duchnowska, Zuzanna Sas, Maciej Zakrzewski, Anita Florkowska, Martyna Nalepa, Aleksandra Skweres, Filip Suchożebski, Alicja Mąka, Jakub Szymanowski, Piotr Michaluk, Katarzyna Winiarska, Maciej Cieśla, Anna Marusiak, Remigiusz A. Serwa, Michał Węgrzynowicz

**Affiliations:** 1 Mossakowski Medical Research Institute, Polish Academy of Sciences, Pawińskiego 5, 02-106 Warsaw, Poland; 2 Doctoral School of Translational Medicine, Centre of Postgraduate Medical Education, Marymoncka 99/103, 01-813 Warsaw, Poland; 3 IMol Polish Academy of Sciences, Flisa 6, 02-247 Warsaw, Poland; 4 Nencki Institute of Experimental Biology, Polish Academy of Sciences, Pasteura 3, 02-093 Warsaw, Poland; 5 Faculty of Biology, University of Warsaw, Miecznikowa 1, 02-096 Warsaw, Poland

**Keywords:** putrescine, polyamines, bioorthogonal probe, neuronal vs. astrocytic uptake, neuropathology

## Abstract

Polyamines regulate multiple cellular pathways, and their intracellular availability is controlled in part by transport. We developed imaging putrescine (iPUT), a clickable putrescine analog expected to retain key properties of native putrescine and enable bio-orthogonal fluorophore conjugation for high-resolution imaging. In MCF-7 cells, iPUT accumulated predominantly in nuclei, unlike the directly fluorophore-conjugated probe BODIPY-PUT, highlighting the value of minimal tagging. In hippocampal co-cultures, iPUT accumulated predominantly in neurons. Neuronal labeling was also more frequent than astrocytic labeling in acute hippocampal slices, contrasting with reported glial storage of spermidine and spermine. Subcellular distribution differed across models, with nuclear enrichment in dividing MCF-7 and predominantly extranuclear localization in mature neurons in hippocampal slices. Mimetic hypoxia and excitotoxicity reduced iPUT accumulation in CA1 but not in CA2/3 neurons, and promoted nuclear redistribution in CA1. Thus, iPUT enables investigation of cell-, region-, and stress-dependent putrescine handling.

## Highlights

• The minimal design of iPUT enables precise determination of Put distribution
• iPUT localizes to neurons, contrasting with the glial accumulation of Spd and Spm
• iPUT can be applied across diverse models highlighting its experimental versatility
• Neuronal stress alters iPUT distribution, supporting its use in CNS disease studies

## Graphical abstract

**Figure:**
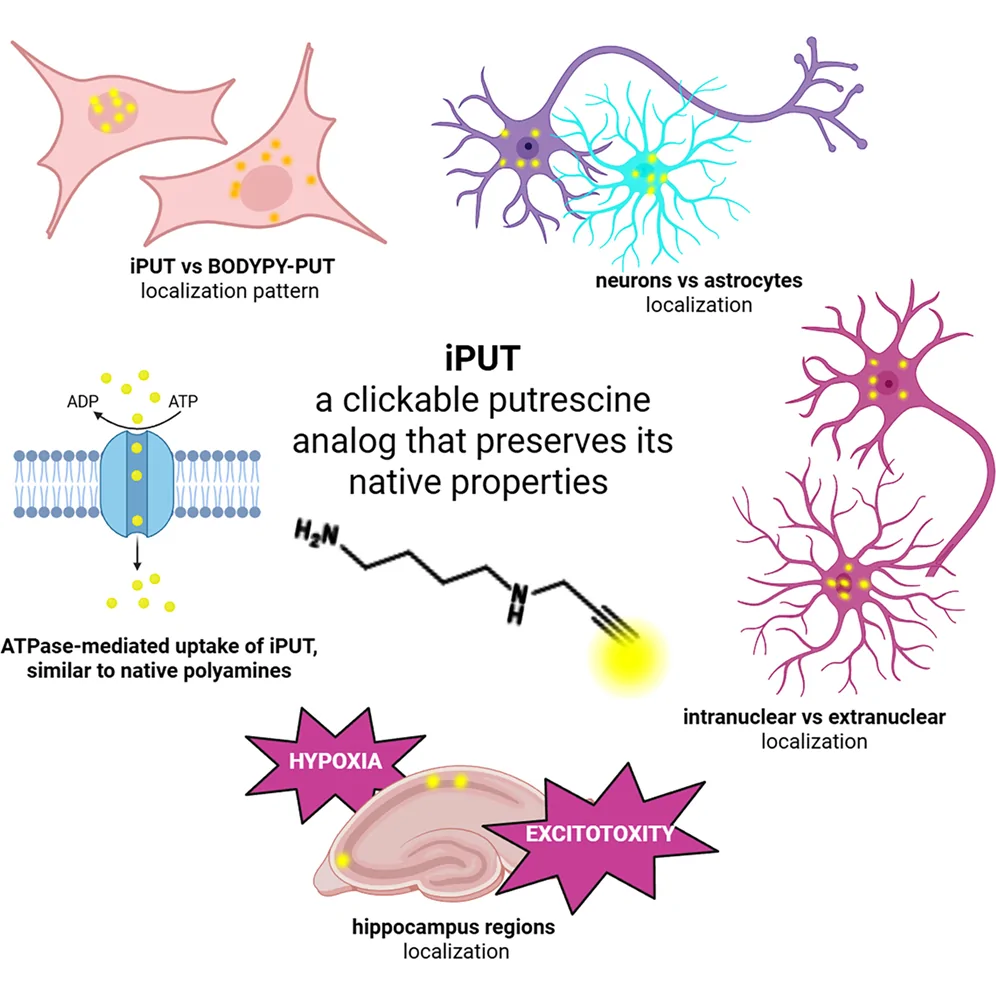

## Teaser

Cell biology; Cellular neuroscience; Neuroscience

## Introduction

Polyamines (putrescine [Put], spermidine [Spd], and spermine [Spm]), are small organic compounds ubiquitously present in cells. Their unique polycationic properties enable them to bind to diverse negatively charged cellular components such as DNA, RNA, acidic proteins, and phospholipids, which allows them to mediate a broad range of cellular functions. Polyamines participate in numerous essential processes, including gene expression, chromatin remodeling, protein synthesis, cell cycle regulation, cellular signaling, and autophagy, among others.^1^^,^^2^ They have emerged as key modulators of cellular plasticity and stress responses, particularly in the nervous system, where they contribute to neuronal differentiation, axonal outgrowth, and synaptogenesis.^3^^,^^4^^,^^5^^,^^6^^,^^7^ They are also significant regulators of synaptic transmission, acting mainly through modulation of ionotropic glutamate receptors (e.g., *N*-methyl-D-aspartate [NMDA] and AMPA) and ion channels.^8^^,^^9^ In pathological context, polyamines have been implicated in both, neuroprotective and neurotoxic mechanisms, and disruption of their homeostasis has been linked to neurodevelopmental,^10^^,^^11^ psychiatric,^12^ and neurodegenerative disorders^13^^,^^14^ as well as to other neurological conditions including epilepsy,^15^^,^^16^ stroke,^17^^,^^18^ and traumatic brain injury.^19^^,^^20^^,^^21^

A deeper understanding of the cellular functions of polyamines requires addressing unresolved questions regarding their subcellular and cellular localization. Earlier studies employing classical biochemical fractionation yielded inconsistent or conflicting results, with polyamines reported to localize predominantly in the nucleus (attributed to their strong binding to nucleic acids),^22^ in the cytosol (as free or loosely bound pools),^23^ or in association with membrane-bound organelles, such as mitochondria, lysosomes, and secretory vesicles.^24^^,^^25^ In the central nervous system (CNS), polyamine distribution was reported to vary significantly between cell types. Glial cells have been identified as the major site of Spd and Spm storage in the adult CNS,^26^^,^^27^^,^^28^^,^^29^ while neurons—the principal site of their synthesis, as Spd synthase and Spm synthase are expressed exclusively in neurons.^30^^,^^31^^,^^32^ In addition, acetylated Spm, a product of Spm degradation, has been found in neurons,^33^ further supporting a functional distinction between neurons and glia in metabolism and cellular handling of higher-order polyamines, and underscoring the importance of polyamine transport for maintaining their proper homeostasis in the brain. In contrast, Put synthesis in the CNS appears less cell-type selective. Its biosynthetic enzyme, ornithine decarboxylase, is widely expressed across CNS cell types, including multiple neuronal populations^34^^,^^35^ and glial cells, such as Müller cells in the retina^28^ as well as cortical and hippocampal astrocytes.^27^^,^^36^ Moreover, polyamine localization at both the cellular and subcellular levels can be dynamically regulated throughout the development, by the metabolic state or external stimuli. Unlike in the adult CNS, Spd was detected in neurons in addition to glial cells in the postnatal rat retina, although neuronal Spd localization was lost within three weeks after birth.^27^ On the other hand, pathological insults such as excitotoxicity^37^^,^^38^^,^^39^ or ischemia^40^^,^^41^ are known to markedly increase ornithine decarboxylase expression and polyamine production.

To precisely determine localization, we employed a Put imaging probe: a bio-orthogonally labeled analog of Put, the simplest naturally occurring polyamine, which we term iPUT (imaging Put). iPUT features a minimal propargyl “click” tag that preserves the compact size and dicationic character of native Put, thereby promoting native-like biomolecular interactions, including cellular uptake and subcellular trafficking. Following an incubation with the probe, iPUT-treated cells or tissue slices are fixed, permeabilized, and subjected to click chemistry^42^^,^^43^^,^^44^ with azido-functionalized fluorescent reporters, allowing visualization of iPUT distribution at subcellular and cellular resolution. We employed iPUT in MCF-7 cell line culture and mixed astrocyte-neuron culture, enabling visualization of predicted subcellular patterns and cell type-specific distribution of Put. We then applied iPUT in acute mouse hippocampal slices to compare Put accumulation across hippocampal subregions and to assess its redistribution in response to neurotoxic stimuli.

## Results and discussion

### Comparison of iPUT and BODIPY-PUT distribution in MCF-7 cells

**Figure 1:**
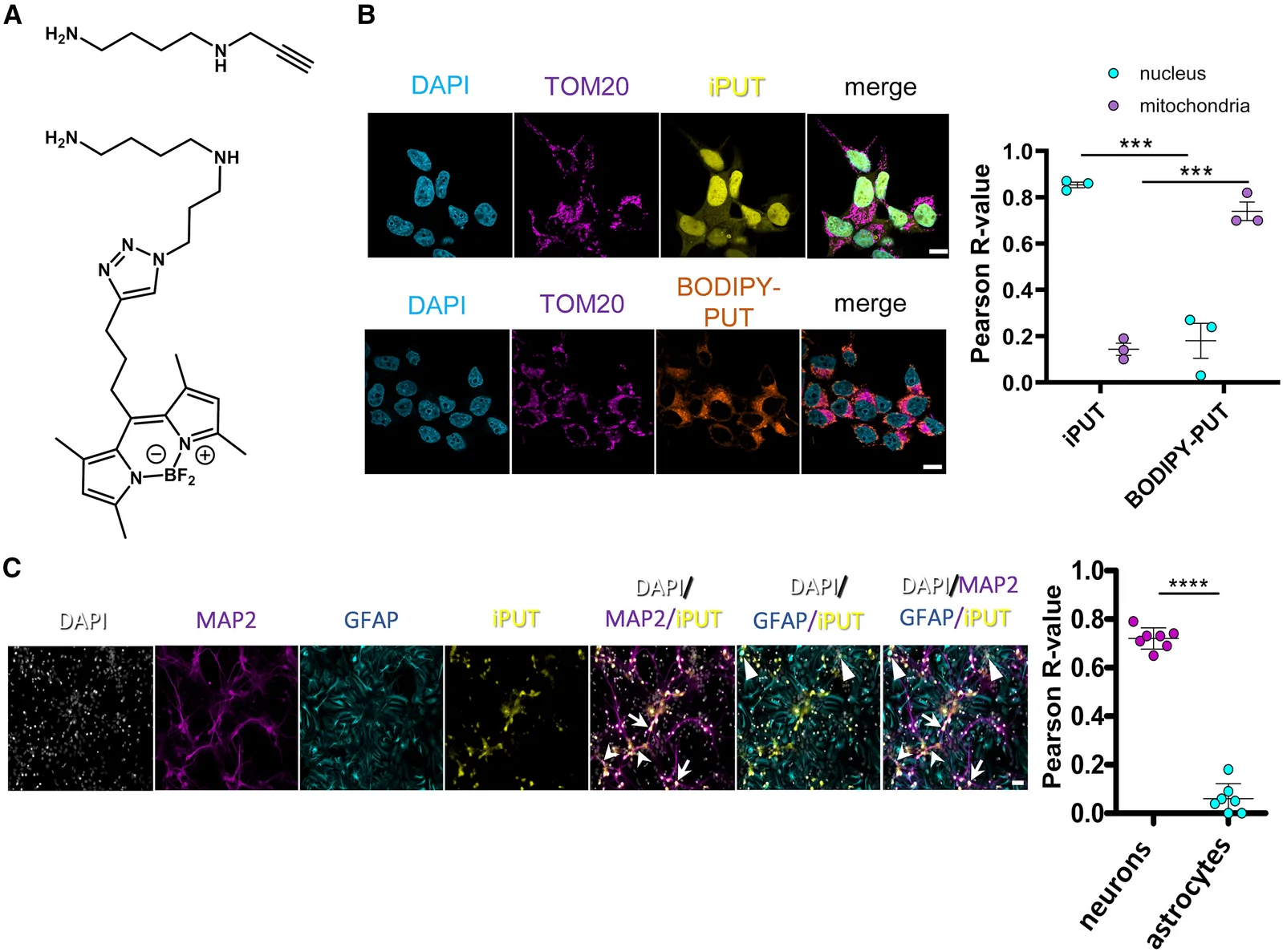
Distribution of Put probes in cell cultures (A) Chemical structures of iPUT and BODIPY-PUT. (B) Distribution of iPUT and BODIPY-PUT in MCF-7 cells. Left: representative confocal images of MCF-7 cells incubated with iPUT (yellow) or BODIPY-PUT (orange) and labeled for mitochondria by TOM20 immunofluorescence (magenta) and nuclei with DAPI (cyan). Scale bars, 10 μm. Right: iPUT/BODIPY-PUT-DAPI and iPUT/BODIPY-PUT-TOM20 colocalization quantification with Pearson’s correlation coefficient (*n* = 3 cultures per experimental condition). (C) Distribution of iPUT in primary mouse hippocampal astrocyte-neuron co-cultures. Left: representative confocal images of co-cultures incubated with iPUT (yellow) and labeled for neurons by MAP2 immunofluorescence (magenta), astrocytes by GFAP immunofluorescence (cyan), and nuclei with DAPI (white). Arrows indicate neuronal bodies with iPUT accumulation within their nuclear and extranuclear compartments. Arrowheads indicate neuronal processes exhibiting iPUT accumulation. Triangles point to astrocytes with iPUT accumulation. Scale bars, 50 μm. Right: iPUT-MAP2 and iPUT-GFAP colocalization quantification with Pearson’s correlation coefficient (*n* = 7 cultures). Data information: in (B) and (C) data are presented as mean ± SEM, ∗∗∗*p* < 0.001, ∗∗∗∗*p* < 0.0001, Student’s *t* test.

We synthesized iPUT (Figure 1A) in four steps, as recently described.^45^ We initially evaluated its performance in the human breast cancer cell line MCF-7, a model in which polyamine transport activity has previously been characterized using BODIPY-polyamine conjugates as surrogates for polyamines.^46^ Alongside iPUT, we treated MCF-7 cells with native Put (negative control, non-fluorescent and lacking a chemical tag, being then incapable of covalent fluorophore labeling via click chemistry) and with a commercially available pre-labeled conjugate (BODIPY-PUT), which can be directly imaged in live or fixed cells by fluorescence microscopy. iPUT vs. BODIPY-PUT imaging revealed striking differences in their subcellular distribution. iPUT, visualized using TAMRA-5-azide, a commercially available fluorescent reporter widely used in bioorthogonal workflows, localized predominantly to the nucleus, with minimal cytoplasmic signal, suggesting efficient nuclear trafficking and retention. In contrast, BODIPY-PUT displayed mainly extranuclear distribution, with strong colocalization with mitochondria (co-labeled with Translocase of the Outer Membrane 20 [TOM20] and minimal nuclear accumulation [Figure 1B]). As expected, no detectable fluorescence was observed in cells treated with native Put and processed for click labeling (Figure S1A), confirming the specificity of the used Put probe. Quantitative image analysis showed a high Pearson correlation between BODIPY-PUT and TOM20 (R = 0.74 ± 0.02), and a very low correlation with nuclear staining (DAPI) (R = 0.18 ± 0.07), in evident contrast to iPUT, which correlated strongly with DAPI (Pearson’s R = 0.85 ± 0.01) but not with TOM20 (R = 0.14 ± 0.043) (Figure 1B). Notably, polyamine levels and transporter activities in cells fluctuate throughout the cell cycle and are closely linked to growth and division, contributing to the heterogeneous signal observed within cells.^47^^,^^48^^,^^49^

The divergent localization profiles of iPUT and BODIPY-PUT likely reflect the impact of probe design on cellular trafficking. BODIPY-PUT carries a bulky, hydrophobic fluorophore with additional charged groups, which can significantly alter its physicochemical properties relative to native Put. These modifications may impede nuclear entry or promote sequestration within cytoplasmic compartments, thereby limiting its utility as a faithful mimetic of the polyamine. Conversely, the minimal, uncharged propargyl tag in iPUT preserves close structural fidelity to native Put, maintaining a native-like physicochemical profile and supporting accurate intracellular behavior. We propose that this underscores the value of structural minimalism in designing functional polyamine mimetics: iPUT’s robust nuclear localization, together with selective subnuclear exclusion, suggests a closer reflection of endogenous Put than bulkier or more extensively modified analogs. Importantly, we also confirmed that under our experimental conditions (probe incubation up to 2 h), iPUT remains overwhelmingly intact and interconversion to N8-propargylspermidine (iSPD) is negligible.^45^

### Distribution of iPUT in mixed neuronal-astroglial co-cultures

Having validated iPUT in MCF-7 line, we next examined its performance in a mixed neuronal-astroglial co-cultures derived from the mouse hippocampus. Polyamines are known to participate in neuron-glia interactions, and their synthesis and distribution are thought to differ substantially between neurons and glia, as noted earlier. However, suitable tools to study their cell-type-specific distribution in the brain have been lacking. We therefore applied iPUT to primary hippocampal co-cultures to determine which cell type more readily takes up exogenous Put. In this experiment, we replaced the previously used TAMRA-5-azide fluorophore with 7-hydroxy-3-azidocoumarin. This substitution not only demonstrated the broader applicability of our method using an alternative excitation/emission range but also provided analytical advantages. Notably, this coumarin derivative is fluorogenic, becoming fluorescent only upon formation of the triazole product in the click reaction, thereby improving the signal-to-noise ratio. Using this configuration, we detected a strong, homogeneous fluorescent signal, primarily localized in neuronal nuclei and somata, and in some cases extending into proximal dendrites, altogether indicating efficient neuronal uptake and retention of iPUT (Figure 1C). In contrast, astrocytes exhibited significantly lower iPUT accumulation (Pearson’s correlation coefficient of iPUT with glial fibrillar acidic protein [GFAP], R = 0.06 ± 0.02) compared with neurons (Pearson’s correlation coefficient of iPUT with microtubule-associated protein 2 [MAP2], R = 0.72 ± 0.02) (Figure 1C). Moreover, while virtually all neurons showed bright fluorescence, iPUT in astrocytes localized predominantly to the nucleus and a subset of astrocytes displayed little or no detectable signal, indicating variability in uptake capacity across astrocyte subpopulations (Figure 1C).

Comparing subcellular distribution of iPUT across cell models used in our experiments confirms that subcellular localization may be regulated by cell state. In continuously dividing MCF-7 cancer cells, iPUT preferentially accumulates in nuclei, whereas in post-mitotic, differentiated neurons, it appears more evenly distributed between nuclei and the extranuclear somatic compartment. Furthermore, the probe reveals a clear neuron-astrocyte divergence in exogenous Put handling: in co-culture, neurons show higher iPUT accumulation than astrocytes, consistent with increased uptake or diminished catabolism/efflux. This finding contrasts with the prevailing view that in the brain, polyamines are synthesized primarily in neurons but accumulate in glial cells.^26^ It is important to recognize, however, that this view rests largely on Spm/Spd-like immunoreactivity experiments^26^^,^^28^^,^^29^^,^^50^ using antibodies raised against Spm or Spd conjugated to bovine serum albumin, an approach introduced nearly 30 years ago that, despite frequent citation, never saw broad adoption. Moreover, this assumption emphasizing astrocytic localization often overlooks the fact that experiments using Spd/Spm antibodies also demonstrated immunoreactivity in neurons, revealing marked region- and neuron type-dependent heterogeneity, with relatively strong signal in the hippocampus.^26^ Unlike iPUT, which reports exogenous Put uptake, such antibodies detect intracellular polyamine pools downstream of Put. Accordingly, our data may signify that neurons exhibit greater Put uptake capacity than astrocytes under basal culture conditions.

Polyamine transporters and their cell-specific distribution remain poorly characterized, although recent analyses indicate the presence of transporters in neurons, including the plasma membrane carrier SLC45A4^51^ and the lysosomal carrier ATP13A2, both involved in cellular polyamine uptake,^52^ with the latter expressed at very high levels in the pyramidal neurons in the hippocampus.^53^ Astrocytic polyamine uptake systems appear to be characterized in more detail, both at the levels of expression and functional analysis,^54^^,^^55^^,^^56^ with the recently identified transporter ATP13A4 shown to play a crucial role in Spd and Spm accumulation in astrocytes, as reported in the preprint by van Veen et al.^57^ To our knowledge, no report directly compared astrocytic vs. neuronal Put accumulation in co-cultures using an exogenously provided probe, although some attempts to compare astrocytic and neuronal uptake and accumulation of polyamines have been made. For example, Masuko et al., using radiolabeled polyamines, compared Spm and Spd uptake kinetics between synaptosomes isolated from rat brain and rat primary astrocyte cultures. Authors demonstrated that V_max_ was similar for Spm in both preparations, but for Spd it was higher in synaptosomes than in astrocytes. Conversely, K_m_ was lower for both Spm and Spd in astrocytes compared with synaptosomes.^58^ These results may suggest that astrocytic transport systems exhibit higher substrate affinity but lower maximal transport capacity than neuronal (specifically synaptic) ones, however, the design of the study does not allow for fully direct comparisons. Polyamine uptake was also analyzed in primary cultures of granule neurons,^59^ and, separately, astrocytes,^55^ both derived from the same brain region (cerebellum), providing a more directly comparable setting for assessing cell type-specific transport. These analyses revealed polyamine type-dependent differences in uptake capacity between astrocytes and neurons. The most pronounced differences were observed for Spm, with astrocytes showing ∼11-fold lower K_m_ and ∼13-fold higher V_max_ compared with neurons, followed by Spd (comparable K_m_ and ∼8-fold higher V_max_), while kinetic parameters for Put were similar (∼1.5-fold higher K_m_ and 2.5-fold higher V_max_ in astrocytes). These data are consistent with astrocytes serving as a major site of higher-order polyamine accumulation in the brain, while suggesting that neurons can effectively compete with astrocytes for Put. Importantly, *in vitro*, the accumulation dynamics may also depend on the stage of culture. It was further reported that in mixed cerebellar cultures, Put-like immunoreactivity shifted over the course of the culture: initially detected in neurons, then in astrocytes, as the culture matured and finally in microglia, reflecting changes in cellular composition.^60^ The discrepancy between the earlier-cited studies and the predominantly neuronal accumulation of iPUT observed in our study may arise from differences in experimental models and biological conditions. Our experiments were performed in primary hippocampal neuron-astrocyte co-cultures, while the cited studies used either separate astrocytic or neuronal cerebellar cultures^55^^,^^59^ or evolving mixed cerebellar cultures,^60^ suggesting that region-specific properties of neurons and/or astrocytes may contribute to distinct Put handling. In addition, co-culture conditions enable direct neuron-astrocyte interactions and intercellular signaling, which may dynamically regulate uptake and retention mechanisms in response to exogenous substrates, in contrast to monocultures or evolving mixed cultures. Importantly, iPUT was designed as a minimal structural analog of Put, differing only by a small propargyl tag, and is therefore expected to closely mimic the physicochemical properties and cellular behavior of native Put, although subtle effects on transport or intracellular interactions cannot be fully excluded. Together, these factors may underlie the observed differences and highlight region- and model-dependent variability in Put uptake preference between astrocytes and neurons.

### Distribution of iPUT in acute mouse hippocampal slices

To further evaluate the utility of our iPUT probe as a tool for investigating Put trafficking dynamics in the adult brain tissue, with respect to regional and cellular compartmentalization, we applied it to *ex vivo* acute mouse hippocampal slices. This model recapitulates a physiologically relevant complexity of the adult brain, maintaining a well-preserved organization of tissue, significantly intact cell-to-cell interactions, as well as native connectomic architecture.

**Figure 2:**
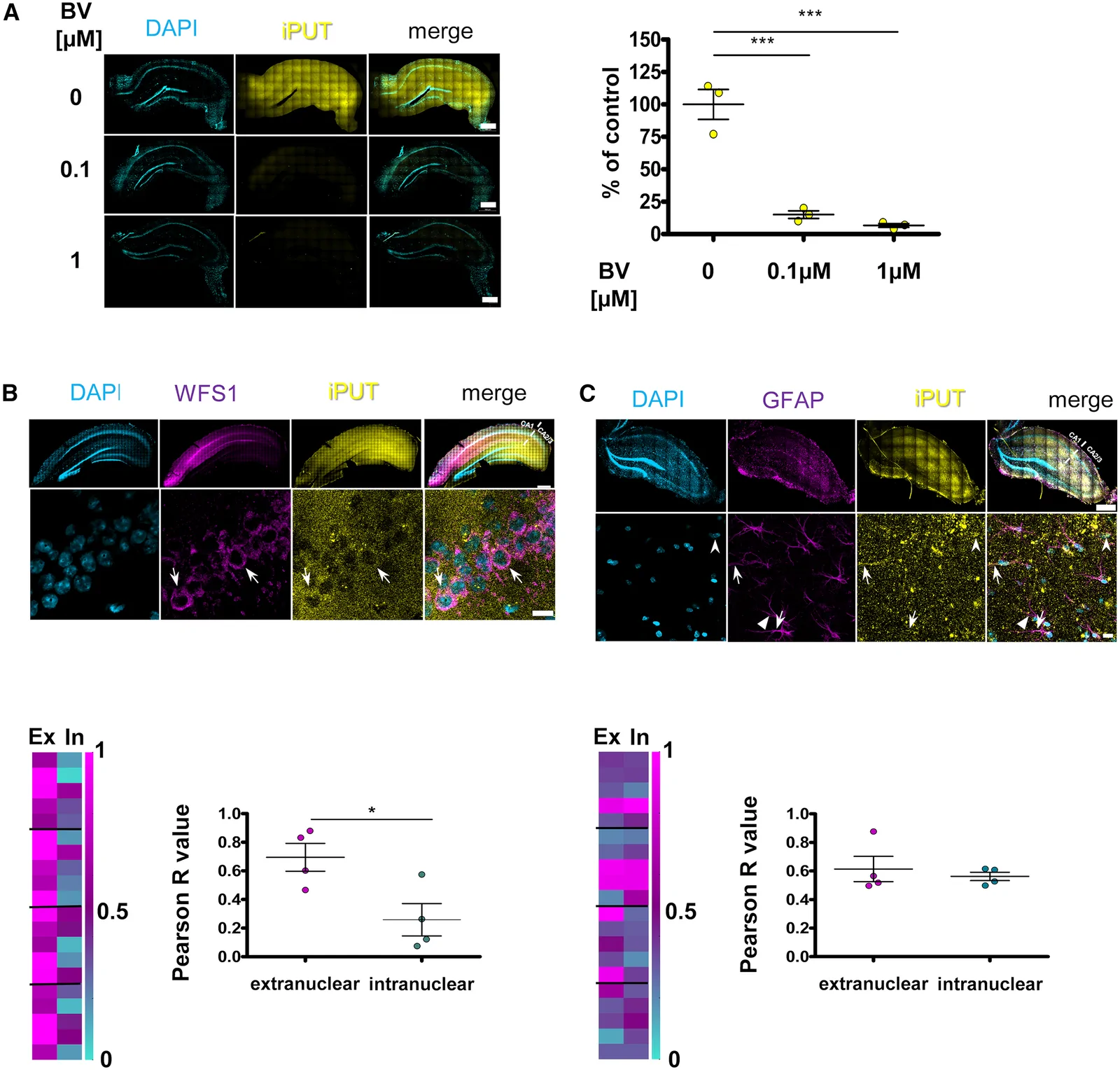
Distribution of iPUT in acute mouse hippocampal slices (A) iPUT accumulation in acute slices in the presence of polyamine uptake inhibitor, BV. Left: representative confocal images of acute hippocampal slices incubated with iPUT (yellow) in the presence of BV. Nuclei are labeled with DAPI (cyan). Scale bars, 500 μm. Right: quantification of mean iPUT fluorescence intensity in slices exposed to iPUT in combination with BV (0, 0.1, and 1 μM) (*n* = 3 animals). (B) iPUT distribution in CA1 pyramidal neurons. Top: representative confocal images of acute slices incubated with iPUT (yellow) and labeled for CA1 pyramidal neurons by WFS1 immunofluorescence (magenta). Nuclei are labeled with DAPI (cyan). Images of whole slices (top) and a fragment of the CA1 stratum pyramidale (bottom) are shown. Arrows indicate CA1 pyramidal neurons exhibiting extranuclear accumulation of iPUT within their somata. Scale bars, 500 μm for low-magnification images, 20 μm for high-magnification images. Bottom left: Pearson’s colocalization heatmaps show iPUT distribution between extranuclear (Ex; labeled with WFS1) and intranuclear (In; labeled with DAPI) compartments within individual neurons. Bottom right: quantification of colocalization of iPUT-WFS1 (indicating extranuclear localization) and iPUT-DAPI (indicating intranuclear localization) with Pearson’s correlation coefficient (*n* = 4 animals). (C) iPUT distribution in CA1 astrocytes. Top: representative confocal images of acute slices incubated with iPUT (yellow) and labeled for astrocytes by GFAP immunofluorescence (magenta). Nuclei are labeled with DAPI (cyan). Images of whole slices (top) and a fragment of the CA1 stratum radiatum (bottom) are shown. Arrowhead indicates an astrocytic nucleus with prominent iPUT accumulation. Arrows indicate astrocytes exhibiting iPUT accumulation within their processes. Triangle points to an astrocyte with negligible iPUT accumulation. Scale bars, 500 μm for low-magnification images, 20 μm for high-magnification images. Bottom left: Pearson’s colocalization heatmaps show iPUT distribution between extranuclear (Ex; labeled with GFAP) and intranuclear (In; labeled with DAPI) compartments within individual astrocytes. Bottom right: quantification of colocalization of iPUT-GFAP (indicating extranuclear localization) and iPUT-DAPI (indicating intranuclear localization) with Pearson’s correlation coefficient (*n* = 4 animals). Data information: in (A), (B), and (C), data are presented as mean ± SEM. (A) Statistical analysis: one-way ANOVA, statistically significant effect of BV treatment, F(2,6) = 55.28, *p* = 0.0001, ∗∗∗*p* < 0.001, post-hoc Bonferroni test. (B and C) ∗*p* < 0.05, Student’s *t* test.

We first tested the specificity of iPUT uptake using benzyl viologen (BV), a potent inhibitor of ATPase-dependent polyamine transport.^46^^,^^61^ While we observed a strong fluorescent signal across all regions and layers of slices treated with iPUT and clicked with TAMRA-5-azide, we found that pre-treatment with either 0.1 or 1 μM BV resulted in a marked depletion of signal intensity, to 15% ± 2.89% and 6.67% ± 1.45%, respectively (Figure 2A). To confirm the technical reliability of our protocol in slices, we also performed negative control experiments, by replacing iPUT with Put or by omitting TAMRA-5-azide in the click reaction. In both conditions, we observed only negligible fluorescence, compared with the positive control (Figure S1B), indicating that in acute slices, the click reaction specifically conjugates TAMRA-5-azide to iPUT and that no significant fluorescence is natively present in the tissue or generated upon incubation with click reaction mixture deprived of the fluorophore. Overall, these results clearly indicate that in our slice model, iPUT faithfully mimics Put with respect to cellular uptake. Moreover, they demonstrate that ATPase-mediated transport constitutes the major pathway for Put entry into hippocampal cells, consistent with the high expression of ATP13A2 reported in pyramidal neurons of the hippocampus.^53^

Having confirmed the specificity of iPUT as a reliable Put probe in acute hippocampal slices, we next analyzed its distribution, focusing on CA1 region, which we immunostained after iPUT labeling, using an antibody against wolframin (WFS1), a protein specifically expressed in CA1 neurons.^62^ We found that iPUT localized in different layers, including the stratum oriens and stratum radiatum, where it displayed a dispersed pattern within the neuropil, as well as in neurons of the stratum pyramidale (Figure 2B). Notably, the subcellular distribution of iPUT in CA1 pyramidal neurons differed from that observed in dissociated cultures: although neuronal nuclei were labeled in slices, the signal was predominantly extranuclear (Figure 2B). Co-localization analysis supported this observation, showing a significantly higher Pearson’s R value for iPUT-WFS1 co-localization (R = 0.69 ± 0.1) than for iPUT-DAPI co-localization (R = 0.26 ± 0.11).

We also observed iPUT retention in a subset of astrocytes distributed across all layers of CA1 region, although astrocytic labeling was less frequent than neuronal labeling. Previously reported comparable model based on rat acute hippocampal slices exposed to biotin-conjugated Spm showed that this Spm probe accumulated predominantly in astrocytes,^63^ highlighting clear differences in cellular specificity of Put vs. Spm uptake. In our model, within astrocytes, the probe was, on average, distributed equally between nuclei and extranuclear compartments, including both cell bodies and processes (Figure 2C). This was supported by co-localization analysis, which showed comparable Pearson’s R values for iPUT-GFAP and iPUT-DAPI (R = 0.61 ± 0.09 and R = 0.56 ± 0.03, respectively) (Figure 2C). Interestingly, while most astrocytes displayed a uniform distribution of the probe, some cells exhibited either predominantly nuclear or predominantly extranuclear signal, suggesting potential cell type-dependent differences in iPUT localization (Figure 2C).

The differences in intracellular iPUT distribution observed between primary cultures and acute slices may again reflect the developmental or physiological state of the cells. Our co-cultures represent developing, immature neurons *in vitro* (already post-mitotic, but not fully mature), whereas slices contain mature neurons in their native tissue environment. This may correspond to the more quiescent state of adult neurons, which no longer extend processes. Instead, they are extensively engaged in neurotransmission, a process in which polyamines play important roles, acting as neuromodulators of NMDA glutamate receptors^8^^,^^64^ and as modifiers of numerous cellular ion channels.^15^^,^^65^ By contrast, neurons in primary cultures, although capable of forming functional synapses, receive and transmit significantly fewer inputs and outputs than neurons *in situ*. At the same time, unlike neurons *in situ*, they continue to undergo extensive growth by extending dendrites and axons, as well as synaptogenesis, processes that are tightly controlled at the level of transcription,^66^ in which polyamines can be significantly involved.^67^^,^^68^ This may explain the increased demand for nuclear polyamine availability to support transcriptional processes, and therefore the enhanced accumulation of iPUT in neuronal nuclei observed in primary cultures compared with acute slices.

### Changes in iPUT distribution in hippocampal CA neurons under neurotoxic stress

**Figure 3:**
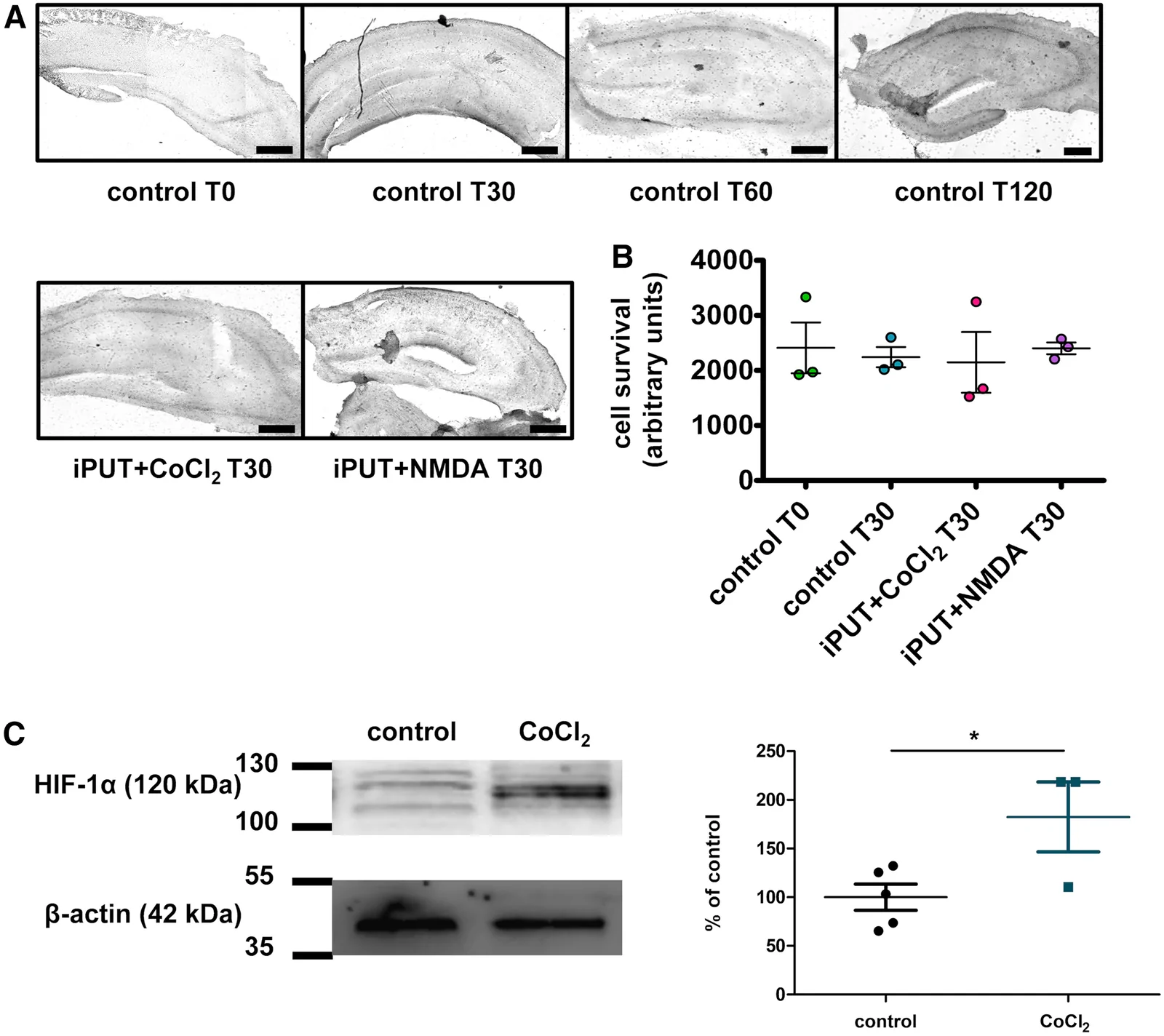
Induction of neurotoxic stress in acute hippocampal slices (A) Trypan blue assay of cell damage following CoCl_2_ or NMDA exposure. Representative bright-field images of trypan blue-stained acute hippocampal slices at baseline, and after 30, 60, and 120 min in control conditions (control T0, T30, T60, T120, respectively), or following 30 min exposure to 500 μM iPUT in combination with 1 mM CoCl_2_ (iPUT + CoCl_2_ T30), or to 500 μM iPUT in combination with 250 μM NMDA (iPUT + NMDA T30). Scale bars, 500 μm. (B) Quantification of CA1 neuron injury at baseline (control T0) and after 30 min incubation in control conditions and in the presence of iPUT with CoCl_2_ and iPUT with NMDA (control T0, control T30, iPUT + CoCl_2_ T30, and iPUT + NMDA T30, respectively) based on CA1 stratum pyramidale trypan blue absorbance measurements (*n* = 3 animals per each experimental condition). (C) Measurements of HIF-1α stabilization following 30 min exposure of acute slices to iPUT with CoCl_2_. Left: representative western blot analysis of protein content of HIF-1α and β-actin (loading control) in acute hippocampal slices incubated for 30 min in the absence or presence of CoCl_2_. Right: densitometric analysis of HIF-1α band normalized to β-actin band (*n* = 5 for control condition, *n* = 3 for iPUT + CoCl_2_ exposure). Data information: (B and C) data are presented as mean ± SEM. (B) Statistical analysis: one-way ANOVA: no effect of treatment, F(3,8) = 0.117, *p* = 0.947. (C) ∗*p* < 0.05, Student’s t test.

Finally, since polyamines are known to be critically involved in both the mediation of and protection against various brain pathologies, we tested whether hippocampal slices respond to stress stimuli by altering the pattern of iPUT accumulation. We induced models of two major brain pathologies—hypoxia, using a mimetic approach in which slices were exposed to CoCl_2_, a treatment that stabilizes hypoxia-inducible factor-1α (HIF-1α), a central regulator of hypoxia-responsive genes,^69^ and excitotoxicity, by exposing slices to NMDA, a potent NMDA receptor agonist. Slices were analyzed for iPUT distribution after 30 min (T30) of exposure to iPUT in combination with either CoCl_2_ or NMDA, a time point at which the trypan blue assay showed no evident cell death in either control slices or those exposed to stress-inducing factors (Figures 3A and 3B). Importantly, in our acute slice model, control slices exhibited no further cell damage after additional 30 min of NMDA treatment (T60) with signs of damage at T120 (Figure 3A), indicating that at T30, the slices were in generally good condition, and therefore suitable for exogenous stress-induced experiments, effectively ruling out a significant pathological component of spontaneous cell damage in this model. Additionally, to confirm the induction of mimetic hypoxia by CoCl_2_, we measured stabilization of HIF-1α, and indeed found that after 30 min of treatment, its levels were significantly elevated (by ∼50%) in slice homogenates (Figures 3C and S2).

**Figure 4:**
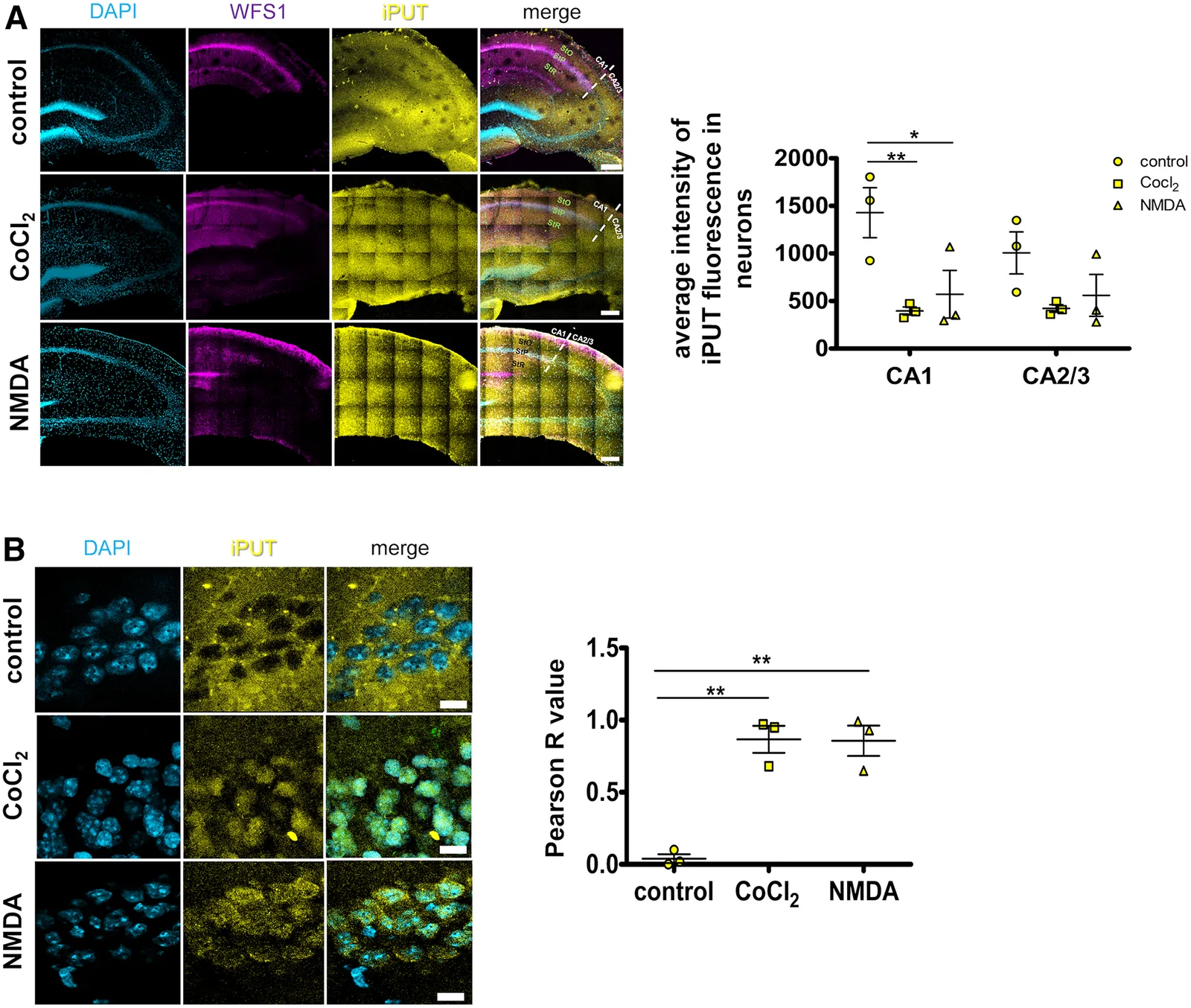
Changes in iPUT distribution in hippocampal CA neurons in acute slices exposed to neurotoxic conditions (A) Changes in iPUT accumulation in pyramidal neurons of CA1 and CA2/3 following exposure to CoCl_2_ or NMDA. Left: representative confocal images of acute slices incubated for 30 min with iPUT (yellow) in control conditions or in combination with CoCl_2_ or NMDA. Slices are labeled for CA1 pyramidal neurons by WFS1 immunofluorescence (magenta). Nuclei are labeled with DAPI (cyan). StO, stratum oriens; StP, stratum pyramidale; StR, stratum radiatum. Scale bars, 500 μm. Right: quantification of mean iPUT fluorescence intensity in CA1 and CA2/3 pyramidal neurons in control slices and in slices exposed to CoCl_2_ or NMDA (*n* = 3 animals). (B) Changes in intracellular localization of iPUT in CA1 neurons following exposure to CoCl_2_ or NMDA. Left: high-magnification representative confocal images of CA1 neurons in acute slices incubated for 30 min with iPUT (yellow) in control conditions or in combination with CoCl_2_ or NMDA. Nuclei are labeled with DAPI (cyan). Scale bars, 10 μm. Right: Pearson’s correlation coefficient quantification of colocalization of iPUT-DAPI (indicating intranuclear localization) in CA1 neurons in slices incubated in control conditions or exposed to CoCl_2_ or NMDA (*n* = 3 animals). Data information: (A and B) data are presented as mean ± SEM. (A) Statistical analysis: two-way ANOVA, no effect of region: F(1,12) = 0.721, *p* = 0.412; statistically significant effect of treatment F(2,12) = 9.517, *p* = 0.003; no interaction F(2,12) = 0.807, *p* = 0.469; ∗*p* < 0.05, ∗∗∗*p* < 0.001, post-hoc Bonferroni test. (B) Statistical analysis: one-way ANOVA, statistically significant effect of treatment: F(2,6) = 32.69, *p* = 0.0006, ∗∗*p* < 0.01, post-hoc Bonferroni test.

Upon treatment with either CoCl_2_ or NMDA, we observed a significant decrease in iPUT accumulation in CA1 neurons, as determined by TAMRA-5-azide intensity quantification following click reaction in WFS1-positive pyramidal neurons (from 1428.97 ± 371.07 a.u. in control slices to 395.08 ± 60.68 and 571.17 ± 353.36 a.u. in slices exposed to CoCl_2_ and NMDA, respectively) (Figure 4A). In contrast, iPUT accumulation in CA2/3 neurons (WFS1-negative) was unaffected by either treatment (Figure 4A), although a trend toward a decrease was visible (1005.15 ± 220.73 a.u. in control slices, 422.13 ± 39.11 a.u. in CoCl_2_-exposed slices, and 595.02 ± 220.11 a.u. in NMDA-exposed slices). This observation is consistent with well-established differences in the vulnerability of hippocampal subfields, with CA1 neurons being highly sensitive to pathological insults, while CA3, and particularly CA2, exhibiting greater resistance, as demonstrated in humans^70^^,^^71^^,^^72^ and in animal models.^70^^,^^73^^,^^74^ Although the cellular pathways underlying these regional differences remain unclear, our findings may suggest that distinct dynamics of polyamine accumulation could potentially contribute to differential sensitivity of hippocampal subregions to excitatory stress-driven pathology.

The involvement of polyamines in neurological disorders is complex and context-dependent, varying with type of pathology, disease stage, individual polyamine, metabolic state, and site of action. Notably, the pathogenic impact of impaired polyamine uptake has recently been evidenced by the identification of ATP13A2 as a lysosomal polyamine transporter.^52^ Loss or mutations of ATP13A2, which cause Kufor-Rakeb syndrome, an early-onset Parkinson’s disease with dementia,^53^ were shown to reduce uptake of polyamines (including Put) and exacerbate the toxicity of extracellular polyamines. While exogenous Put, in contrast to Spd or Spm, did not induce neuronal death in this model,^52^ our data raise the possibility that diminished iPUT accumulation in CA1 following CoCl_2_ or NMDA exposure reflects a general impairment of polyamine uptake under stress. Because ATP13A2 appears to be the major polyamine transporter in hippocampal pyramidal neurons,^53^ a stress-induced reduction in CA1 neuronal polyamine uptake could contribute to the region’s increased vulnerability to pathology in comparison with CA2/3. However, this hypothesis requires further investigation.

Finally, we analyzed the effect of CoCl_2_ or NMDA exposure on the intracellular distribution of iPUT in CA1 neurons and found that both treatments markedly increased nuclear accumulation of the probe (Pearson’s iPUT-DAPI correlation coefficient R = 0.04 ± 0.04, R = 0.87 ± 0.13, and R = 0.86 ± 0.15 for control, CoCl_2_, and NMDA, respectively; Figure 4B). This suggests that Put in CA1 neurons may increase Put involvement in the regulation of gene expression under stress conditions. How this redistribution contributes to neuronal impairment remains an unanswered question. It may reflect an attempt to activate neuroprotective pathways by adapting gene expression to pathological stress. Alternatively, it could contribute to pathology by reducing the availability of Put for its cytoplasmic functions. This possibility may be particularly relevant given the established role of polyamines in regulation of ion channels.^8^^,^^9^ Because ion imbalance represents a rapid neuronal stress response and a key mediator of pathways leading to neuronal dysfunction and death, the translocation of Put to the nucleus may deprive ion channels of an important regulatory mechanism, thereby contributing to uncontrolled ion influx to the neurons and aberrant fluxes between cell compartments.

### Summary and conclusions

In summary, we present iPUT—a clickable Put probe minimally modified with a propargyl tag to preserve the properties of native Put. iPUT was efficiently taken up by cells in a cancer cell line, mixed primary neuron-astrocyte cultures, and acute hippocampal slices, and could be visualized at subcellular resolution. Its versatility was demonstrated by labeling with two fluorophores and compatibility with immunofluorescence, enabling precise localization across tissue, cellular, and subcellular contexts. By applying iPUT, we show that Put localization depends on cell type, cell state, and pathophysiological condition. Notably, contrary to the established pattern of Spd/Spm cellular distribution, neurons accumulated considerably more iPut than astrocytes. Under stress, CA1, but not CA2/3 pyramidal neurons exhibit reduced accumulation capacity, a change that may contribute to their higher vulnerability to injury. In response to a noxious stimulus, CA1 neurons also shifted their iPUT pool from extra-to intranuclear compartment, indicating either an adaptive protective response or activation of injury pathways. Unlike a covalently fluorophore-conjugated Put probe (BODIPY-PUT), iPUT displays a distinct distribution pattern, retaining native Put behavior and reflecting the disruptive effect of bulky fluorophore in BODIPY-PUT. We propose iPUT as a practical, advantageous tool for investigating cellular Put dynamics, offering new insights into the biological functions of this underexplored metabolite and polyamines more broadly.

### Limitations of the study

Although the minimal design of the iPUT is expected to minimize the perturbation relative to native Put, it has not been experimentally established whether iPUT faithfully reproduces the subcellular distribution of newly imported Put. A direct side-by-side comparison of the intracellular localization of iPUT and native Put following uptake within the same time window would be technically challenging. In principle, this might be addressed using mass spectrometry imaging of stable-isotope-labeled tracers. However, such an approach would likely require substantial methodological development, and we were unable to identify a published precedent demonstrating successful high-resolution subcellular imaging of newly imported Put. In addition, the models used in the study could be complemented by an *in vivo* model in which blood-brain barrier permeability would be assessed as a critical property for applications in central nervous system research. Finally, to explore the importance of hippocampal region-specific Put accumulation and intracellular dynamics for neuronal resistance to injury, a model of differential susceptibility of CA1 vs. CA2/3 neurons to cell death could be used to track, in a spatiotemporal manner, how iPUT accumulation correlates with neuronal damage.

## Resource availability

### Lead contact

Requests for further information and resources should be directed to and will be fulfilled by the lead contact, Michał Węgrzynowicz (mwegrzynowicz@imdik.pan.pl).

### Materials availability

The chemical probe iPUT will be made available on a collaborative basis by Remigiusz A. Serwa (r.serwa@imol.institute).

### Data and code availability

• The raw data used to generate the figures in this article have been deposited in Mendeley Data (version 1), https://doi.org/10.17632/mvjdt9xndt.
• This paper does not report original code.
• Any additional information required to re-analyze the data reported in this paper are available from the lead contact upon request.

## Acknowledgments

This work was supported by the 10.13039/501100004442National Science Centre, Poland (grant nos. 2023/07/X/NZ4/00420 [to A.O.], 2020/38/E/ST4/00250 [to R.A.S.], and 2023/49/N/NZ4/02660 [to A.S.]) and the Mossakowski Medical Research Institute, 10.13039/501100004382Polish Academy of Sciences (internal research fund no. FBW-019 [to M.W.]).

## Author contributions

A.O., conceptualization, formal analysis, funding acquisition, investigation, methodology, project administration, supervision, validation, visualization, writing – original draft, and writing – review and editing; A.D., investigation; Z.S., investigation, methodology, and formal analysis; M.Z., investigation, methodology, formal analysis, and writing – review and editing; A.F., investigation, methodology, and formal analysis; M.N., investigation and writing – review and editing; A.S., funding acquisition, investigation, and writing – review and editing; F.S., investigation; A. Mąka, investigation; J.S., visualization; P.M., investigation and writing – review and editing; K.W., investigation, resources, and writing – review and editing; M.C., resources and writing – review and editing; A. Marusiak, supervision and writing – review and editing; R.A.S., conceptualization, funding acquisition, methodology, project administration, resources, supervision, writing – original draft, and writing – review and editing; M.W., conceptualization, funding acquisition, methodology, project administration, resources, supervision, writing – original draft, and writing – review and editing.

## Declaration of interests

The authors declare no competing interests.

## Declaration of generative AI and AI-assisted technologies in the writing process

During the preparation of this work, the authors used OpenAI ChatGPT 4.0 to improve the language and readability. After using this tool, the authors reviewed and edited the content as needed and take full responsibility for the content of the published article.

## STAR★Methods

### Key resources table

**Table:** 

REAGENT or RESOURCE	SOURCE	IDENTIFIER
**Antibodies**		

Anti-WFS1	STI	Cat# 26995-1-AP-SS; RRID: AB_2880717
Anti-GFAP	Abclonal	Cat# A0237; RRID: AB_2757050
Anti-rabbit, biotinylated	Vector Laboratories	Cat# BA-1000; RRID: AB_2313606
Streptavidin, Alexa Fluor 488-conjugated	Invitrogen	Cat# S11223; RRID: AB_2315383
Anti-HIF-1α	Thermo	Cat# PA1-16601; RRID: AB_2117128
Anti-β-actin, HRP-conjugated	Abcam	Cat# ab20272; RRID: AB_445482
Secondary anti-rabbit, HRP-conjugated	Sigma	Cat# 12-348; RRID: AB_390191
Anti-TOM20	Proteintech	Cat# 11802-1-AP; RRID: AB_2207530
Secondary goat anti-rabbit, Alexa Fluor 488-conjugated	Invitrogen	Cat# A-11008; RRID: AB_143165
Anti-MAP2	Merck	Cat# Ab5622; RRID: AB_91939
Anti-GFAP, Cy3-conjugated	Merck	Cat# C9205; RRID: AB_476889

**Chemicals, peptides, and recombinant proteins**		

NaCl	Merck	S9888
KCl	Chempur	11397402
HEPES	Polaura	PA0330868
MgCl_2_	Chempur	116120500
Glucose	Chempur	114595602
PBS tablets	Gibco	18912-014
Tween 20	Sigma	102636
Nonidet P-40	Sigma	I8896
Sodium deoxycholate	Sigma	30970
SDS	Chempur	151213
Tris-HCl	Sigma	T15760
Glycerol	Sigma	SHBS2744
Bromophenol blue	Sigma	B0126
β-mercaptoethanol	Sigma	M3148
Put (putrescine)	Polaura	PA-0398739
NMDA	Sigma	M3262
CoCl_2_	Sigma	7646-79-9
Benzyl viologen (BV)	Sigma	271845
Methanol (analytical grade)	Supelco	1.06007.2500
Dimethyl sulfoxide (DMSO)	VWR	67685
CuSO_4_	Sigma	209198
THPTA	Sigma	762942
TAMRA-5-azide	Jena Bioscience	CLK-FA008-1
Normal Horse Serum (NHS)	Vector Laboratories	S-2000
FluoroSafe mounting medium	Merck	345789
HBSS (no Ca^2+^/Mg^2+^)	Merck	H6648
Sodium Pyruvate	Merck	S8636
DMEM	Thermo Fisher Scientific	31966-021
MEM	Thermo Fisher Scientific	51200-046
FBS	Merck	F4135
Albumin	Bioshop	ALB001.250
Trypsin Inhibitor	Merck	T9253
Papain	Worthington	LS003126
Deoxyribonuclease I	Worthington	LS002007
Poly-D-lysine	Merck	P7886-100MG
Laminin	Merck	11243217001
Neurobasal-A (no phenol red)	Thermo Fisher Scientific	12349-015
B-27 Supplement	Thermo Fisher Scientific	17504044
GlutaMAX	Thermo Fisher Scientific	35050-061
DRAQ5	–	–
Fluorescent mounting medium	DakoCytomation	–
Protease Inhibitor Cocktail	Sigma	P8340
BCA Protein Assay Kit	Millipore	71285-3
Nitrocellulose membrane 0.2 μm	Amersham	GE10600001
SuperSignal West Dura	Thermo	34075
Trypan Blue	Thermo Fisher Scientific	15250-061
EDTA	Polaura	PA-03-4011-E
acryl:bis	VWR	97064-750
APS	Chempur	11391908
TEMED	sigma	110-18-9
glycine	sigma	56-40-6
Tris base	sigma	77-86-1
trypan blue	sigma	72-57-1

**Deposited data**		

Raw data	Mendeley Data	https://doi.org/10.17632/mvjdt9xndt

**Experimental models: Cell lines**		

MCF-7	N/A	RRID:CVCL_0031

**Experimental models: Organisms/strains**		

C57BL/6J	MMRI PAS in-house breeding colony	RRID: MGI:3028467
C57BL/6J	NIEB PAS in-house breeding colony	RRID: MGI:3028467

**Software and algorithms**		

ImageJ/Fiji	1.54g	NIH
ZEN 2012 sp5 Black edition	14.0.9.201	Zeiss
ZEN Blue	3.6.095.00000	Zeiss
GraphPad Prism	10.6.0	GraphPad Software
Chemidoc software	3.0.1	Bio-Rad

### Experimental model and study participant details

#### Ethic statement

Direct approval from the Local Ethics Committee was not required for the experiments reported in this study, as all data were obtained from tissues collected from animals that had not undergone any *in vivo* experimental procedures. According to the Act of 15 January 2015 on the protection of animals used for scientific or educational purposes, sacrificing an animal solely for the purpose of collecting organs or tissues for the purposes specified in Article 3 of the Act (including scientific research) is not regarded as an experimental procedure and therefore does not require ethical approval, provided that euthanasia is performed using a method listed in Annex IV of Directive 2010/63/EU by personnel holding appropriate authorization.

In this project, animals from which tissues were collected were euthanized either by cervical dislocation (adult mice) or decapitation (newborn pups), both of which are methods listed in Annex IV of Directive 2010/63/EU. All activities were carried out by experienced personnel holding appropriate certificates issued by the Polish Laboratory Animal Science Association (PolLASA) as well as institutional authorization permitting mouse euthanasia and tissue collection for experimental purposes using methods compliant with Annex IV of Directive 2010/63/EU.

#### Animals

C57BL/6J mice (RRID: MGI:3028467) were used in this study. Animals used to establish primary hippocampal neuron–astrocyte co-cultures were obtained from the Animal Facility of the Nencki Institute of Experimental Biology, Polish Academy of Sciences, and animals used for acute slice preparation were obtained from the Animal Facility of Mossakowski Medical Research Institute, Polish Academy of Sciences. In both facilities mice were bred and maintained in-house under a 12 h light/dark cycle, with *ad libitum* access to food and water.

#### MCF-7 cell line

MCF-7 (RRID:CVCL_0031) human breast cancer cells were cultured in Dulbecco’s Modified Eagle Medium (DMEM; Capricorn) supplemented with 10% fetal bovine serum (FBS; Capricorn) and 1% penicillin-streptomycin solution (Gibco). Cells were cultured in a humidified incubator at 37°C with 5% CO_2_. Short Tandem Repeats analysis was not performed. Cells were routinely tested for mycoplasma infection and found to be negative.

#### Primary hippocampal neuron–astrocyte co-cultures

The cultures were prepared from P0 (gender not confirmed) mouse pups. Independent cultures were prepared from a single litter. Before decapitation, the pups were anesthetized on ice. Next, the hippocampi were isolated in an ice-cold HBSS without Ca^2+^ and Mg^2+^ (Merck) supplemented with 1 mM sodium pyruvate (Merck), 5.6 mM Glucose and 10 mM HEPES pH 7.4. The hippocampi were digested for 20 min at 37°C in DMEM (Thermo Fisher Scientific) supplemented with 0.2 mg/mL cysteine, 1 mM CaCl_2_, 0.5 mM EDTA, 15 U/mL papain (Worthington) and 250 U/mL deoxyribonuclease I (Worthington). The digestion reaction was stopped by incubating hippocampi for 5 min at room temperature (RT) in Inactivating Solution prepared in MEM (Thermo Fisher Scientific), supplemented with 10% FBS (Merck), 2.5 mg/mL albumin (Bioshop), and 2.5 mg/mL trypsin Inhibitor (Merck). Tissue was rinsed 3 times with MEM supplemented with 10% FBS, 1 mM sodium pyruvate, 0.5 mM GlutaMAX, ant then triturated by pipetting in the same medium. The cells were counted in 1:1 dilution of 0.4% trypan blue (Thermo Fisher Scientific) solution, and plated at density 120 000 cells in a 150 μL medium drop placed on 18-mm-diameter coverslip (Assistent, Germany, #1.5) coated with 1 mg/mL poly-D-lysine (Merck) and 2.5 μg/mL laminin (Merck). Cells were incubated for 1 h at a humidified cell culture incubator at 37°C to facilitate their attachment to the coverslips. Next, 1 mL of Maintenance Medium was added, consisting of Neurobasal-A without phenol red (Thermo Fisher Scientific), 2% B-27 supplement (Thermo Fisher Scientific), 0.5 mM GlutaMAX (Thermo Fisher Scientific), and 25 μM β-mercaptoethanol. The next day 0.5 mL of the Maintenance Medium was removed and 1 mL of fresh medium was added. A half of the Maintenance Medium was changed twice a week.

#### Mouse acute hippocampal slices

Adult, 10-12 week-old males were used for the experiments. Following sacrifice, brains were rapidly removed and immersed in ice-cold nutrient buffer (NB; 140 mM NaCl, 4 mM KCl (Chempur), 10 mM HEPES (Polaura), 1 mM MgCl_2_ (Chempur), 10 mM glucose (Chempur); pH 7.4). Coronal sections (100 μm) containing the proximal and medial hippocampus were prepared using a vibratome (Vibroslice 752M, Campden Instruments, England), collected in NB and pre-incubated for 20 min at 37 °C.

Sample size was 3–5 animals per group. Group sizes were *a-priori* calculated based on previous experience with this model and comparable published studies. Animals were randomly assigned to treatment groups. The experimenter was blinded to the treatment during data analysis.

### Method details

#### Chemical synthesis of iPUT

iPUT was prepared as recently described.^45^

#### Probe delivery

##### Cell cultures

Serum-free cell culture media were supplemented with iPUT (75 μM for 2 h in MCF-7 cells and 100 μM for 30 min in neuron-astrocyte cocultures), while negative control cells were incubated with unmodified Put (concentration and time matched to iPUT incubations). Alternatively, MCF-7 cells were incubated with 5 μM BODIPY-PUT (Sigma-Aldrich) for 2 h in serum-free medium.

##### Acute slices

Slices were incubated at 37°C for 30 min with 500 μM iPUT or, as negative controls, with 500 μM native Put (Polaura). To verify the dependence of iPUT uptake on the polyamine transport system, slices were pre-treated with BV (Sigma-Aldrich), an inhibitor of ATPase-dependent polyamine transport, at 0.1 or 1 μM for 20 min prior to iPUT incubation. To induce neurotoxic stress, slices were exposed to 250 μM NMDA (Sigma-Aldrich) to model excitotoxicity or to 1 mM CoCl_2_ (Sigma-Aldrich) to induce mimetic hypoxia by HIF-1α stabilization.^69^ NMDA or CoCl_2_ were added to the NB together with iPUT and incubation was performed for 30 min at 37°C.

#### Click reaction

##### Cell cultures

Following incubation with iPUT, cells were washed twice with phosphate-buffered saline (PBS; Gibco; 2 × 5 min), and fixed with 4% paraformaldehyde (PFA; Sigma-Aldrich) in PBS, at RT for 10 min, and subsequently permeabilized with 0.05% Triton X-100 (Sigma-Aldrich) in PBS, at RT for 3 min. Cells were then incubated at RT for 1 h with a click-labelling mixture: consisting of 0.1 mM TAMRA-5-azide (MedChemExpress) for MCF-7 cells or with 7-hydroxy-3-azidocoumarin (Sigma-Aldrich) for astrocyte-neuron co-cultures and 1 mM CuSO_4_, 1 mM tris(2-carboxyethyl)phosphine (TCEP; Thermo Fisher Scientific), and 0.2 mM tris[(1-hydroxy-propyl-1H-1,2,3-triazol-4-yl)methyl]amine (THPTA; Ambeed) in PBS. Finally, cells were rinsed twice with PBS (2 × 5 min).

##### Acute slices

After treatment, slices were washed for 15 min in NB and fixed in 98% methanol (Supelco) at −20°C ON. The following day, the click reaction was performed in PBST (PBS containing 0.1% Tween 20 (Sigma-Aldrich)) supplemented with 0.1 mM TAMRA-5-azide (Jena Bioscience), 1 mM CuSO_4_ (Sigma-Aldrich), 1 mM TCEP, and 0.2 mM THPTA (Sigma-Aldrich) for 30 min at 37°C. The reaction was quenched by the addition of 500 μM ethylenediaminetetraacetic acid (EDTA) for 10 min at 37°C. Unbound fluorophore was removed by washing slices three times in PBST containing 10% dimethyl sulfoxide (VWR), followed by two additional washes in PBST to minimize potential fluorescence quenching.

#### Immunofluorescence

##### Cell cultures

Nonspecific antibody-binding sites were blocked by incubation in 3% bovine serum albumin (BSA) in PBS for 30 min at RT. Cells were then incubated ON at 4°C with the following primary antibodies diluted in 0.5% BSA in PBS: anti-MAP2 (1:500), anti-GFAP-Cy3-conjugated (1:1000), or anti-TOM20 (1:250). Subsequently, cells stained for MAP2 and TOM20 were incubated with Alexa Fluor 488-conjugated secondary antibody, diluted at 1:200 in 0.5% BSA in PBS at RT for 2 h. To visualize nuclei, cells were incubated with DAPI (for samples stained with iPUT-TAMRA-5-azide or BODIPY-PUT-TAMRA-5-azide) or DRAQ5 (for samples stained with iPUT-7-hydroxy-3-azidocoumarin) diluted 1:1000 in PBS for 5 min at RT. Finally, specimens were mounted with a fluorescent mounting medium (DakoCytomation). The specificity of primary antibodies was confirmed by incubation of samples with secondary antibodies only.

##### Acute slices

Slices were blocked in 5% normal horse serum (NHS; Vector Laboratories) in PBST for 30 min at 37°C. Incubation with rabbit primary antibodies (anti-WFS1, anti-GFAP, diluted at 1:200 in PBST +5% NHS) was performed for 1 h at 37°C. After washing, sections were incubated with a biotinylated anti-rabbit secondary antibody (1:1000) for 1 h at 37°C, followed by incubation with Alexa Fluor 488-streptavidin (1:1000) for 1 h. DAPI (1:10 000) was added during the final 10 min of incubation. Slices were mounted using FluoroSafe mounting medium (Merck).

#### Western blotting

Hippocampal slices were homogenized in RIPA buffer (50 mM Tris-HCl (Sigma-Aldrich), pH 8.0; 150 mM NaCl (Sigma-Aldrich); 1% Nonidet P-40 (Sigma-Aldrich); 0.5% sodium deoxycholate (Sigma-Aldrich); 0.1% sodium dodecyl sulfate (SDS; Chempur)) supplemented with a protease inhibitor cocktail (Sigma-Aldrich) followed by centrifugation at 14 000 × g for 20 min at 4°C (Mikro 220R centrifuge). Protein concentrations were determined using the BCA Protein Assay Kit (Millipore). Equal amounts of protein (15 μg) were denatured in loading buffer (6% SDS, 30% glycerol (Sigma-Aldrich), 0.187 M Tris, 0.033% bromophenol blue (Sigma-Aldrich), 15% β-mercaptoethanol (Sigma-Aldrich)) for 10 min at 95°C and separated on a polyacrylamide gel composed of 4% stacking gel (0.12 M Tris-HCl, 4% acryl:bis, 0.1% SDS, 0.05% ammonium persulfate (APS), 0.12% N,N,N′,N′-Tetramethylethylenediamine (TEMED), pH 6.8) and 12% resolving gel (0.37 M Tris, 12% acryl:bis, 0.1% SDS, 0.05% APS, 0.05% TEMED, pH 8.8). Following electrophoretic separation in a running buffer (191 mM glycine, 24.7 mM Tris base, 0.1% SDS) at 120 V, using a Mini-Protean system (Bio-Rad, USA), proteins were transferred onto 0.2 μm nitrocellulose membrane (Amersham) by wet transfer (0.3 A, 1.5 h) in a transfer buffer (25 mM Tris, 200 mM glycine, 10% methanol). Membranes were blocked in 5% skim milk in Tris-buffered saline with Tween (TBST; 20 mM Tris-HCl, 150 mM NaCl, 0.1% Tween 20) for 30 min at RT, and then incubated ON at 4°C with anti-HIF-1α (1:1000) or anti-β-actin-HRP (1:25000) antibodies diluted in TBST. After washing in TBST, membranes were incubated with HRP-conjugated anti-rabbit secondary antibody (1:10 000) for 1 h at RT do detect HIF-1α. The signal was developed using SuperSignal West Dura substrate (Thermo Fisher Scientific).

#### Trypan blue assay in acute slices

Slices from various experimental conditions were incubated for 3 min in 0.4% trypan blue solution at RT. After incubation, the tissue was washed here times for 2 min each in PBST to remove excess dye. The samples were then fixed in methanol at −20°C for 20 min, mounted on microscope slides and coverslipped using FluoroSafe mounting medium.

### Quantification and statistical analysis

#### Western blotting

Blots were visualized with a Chemidoc Imaging System (Bio-Rad) and band intensities were quantified using ImageJ software. The data were normalized to actin, which was simultaneously probed in each sample as a loading control.

#### Confocal microscopy and image acquisition

##### MCF-7 cell line

Fluorescent images were acquired using a Zeiss LSM 910 confocal microscope with a 63× oil objective (NA 1.4) and GaAsP detectors. DAPI, AF488, and TAMRA were excited at 405, 488, and 561 nm, respectively, and emission was collected at 400–540 nm, 410–545 nm, and 530–700 nm. The pinhole was set to 1 Airy unit. Imaging was performed in frame mode with bidirectional scanning (scan speed 4), zoom 0.9, 6.37 μs pixel dwell time, and no averaging. Parameters were kept constant within experiments.

##### Primary hippocampal neuron–astrocyte co-cultures

Images were acquired using an Axio Observer.D1 microscope (Zeiss) equipped with a 20×/0.4 objective (NA 0.4) and an Axiocam 506 camera. TexRed, EGFP, DAPI, and DRAQ5 were detected using standard filter sets (Ex/Em: 533–558/570–640 nm; 450–490/500–550 nm; 370–410/430–470 nm; 625–655/665–715 nm, respectively). LED illumination (20% intensity) was used for all channels. Exposure times were 4.4 s (TexRed), 1.75 s (EGFP), 19.5 s (DAPI), and 4.4 s (DRAQ5). Acquisition parameters were kept constant within experiments.

##### Acute slices

Whole-slice imaging was performed using an Axio Observer Z1 microscope (Zeiss, Oberkochen, Germany) equipped with a Plan-Apochromat 20×/0.8 objective (NA 0.8) and an EMCCD Rolera EM-C^2^ camera. DAPI, TAMRA, and Alexa Fluor 488 were detected using standard fluorescence filter sets with excitation/emission maxima of 353/465 nm, 543/567 nm, and 493/517 nm, respectively. Exposure times were 200 ms (DAPI and TAMRA) and 70 ms (AF488). Images were acquired without compression and with 1 × 1 binning.

High-resolution images for colocalization analyses were acquired using a laser-scanning confocal microscope (LSM 780, Zeiss) mounted on an Axio Observer platform and equipped with a Plan-Apochromat 63×/1.4 oil immersion objective (NA 1.4). Fluorophores were excited at 405 nm (e.g., DAPI), 488 nm (e.g., Alexa 488), and 561 nm (e.g., TAMRA), and emission was collected at 410–514 nm, 490–614 nm, and 570–659 nm, respectively. The pinhole was set to ∼1 Airy unit, and images were acquired in frame mode with unidirectional scanning and line averaging of 2.

Trypan blue assay imaging was performed in transmitted light mode using Axio Observer Z1 microscope (Zeiss, Oberkochen, Germany) equipped with a Plan-Apochromat 20×/0.8 objective (NA 0.8) and an EMCCD Rolera EM-C^2^ camera. Acquisition parameters were kept constant within each experiment.

#### Image analysis

##### MCF-7 cell lines

ImageJ software was used to quantify the percentage of positively associated (colocalized) pixels between iPUT or BODIPY-PUT signals and markers of mitochondria (TOM20) or nuclei (DAPI). The Pearson’s correlation coefficient (R value) was calculated using Coloc2 plugin for ImageJ, where values close to 1 indicate strong colocalization, and values close to 0 indicate weak colocalization. Each analysis was performed in at least three independent biological replicates.

##### Primary cell culture

ImageJ software was used to quantify the percentage of positively associated (colocalized) pixels between iPUT and neuronal (MAP2) or astrocytic (GFAP) markers in mixed primary cultures, based on randomly selected fields of view.

##### Acute slices

All images were saved in.czi format and processed using ZEN Blue software (Zeiss) and ImageJ (NIH). Mean transmitted light intensity in trypan blue images was quantified using the Histo tool in ZEN Blue within manually drawn regions of interest (ROIs) for each slice. Mean fluorescence intensity of iPUT was measured using the Histo tool in ZEN Blue after manually drawing the ROI, using standardized fluorescence intensity thresholds. Pearson’s correlation coefficients were calculated using the Coloc2 plugin in ImageJ to assess iPUT-WFS1 and iPUT-DAPI colocalization in the cell bodies of CA1 pyramidal neurons, as well as iPUT-DAPI and iPUT-GFAP colocalization in CA1 astrocytes across all hippocampal layers. At least 5 randomly selected ROIs per cell type (neurons or astrocytes) were analyzed (*n* = 4 biological replicates; independent animals).

#### Statistics

All quantitative data are presented as mean ± SEM. Pearson’s correlation coefficients were calculated as described above. Statistical comparisons were performed using Student’s *t* test, or one-way or two-way ANOVA followed by the Bonferroni post-hoc test. Statistical significance was defined as *p* < 0.05 (∗), *p* < 0.01 (∗∗), *p* < 0.001 (∗∗∗), and *p* < 0.0001 (∗∗∗∗). Information on the statistical tests and significance levels for individual datasets is provided in the corresponding figure legends.
