# Supplementary material for: N-propargylputrescine as a minimal-tag probe for imaging putrescine dynamics in cellular homeostasis and stress
Source: iScience. 2026 Sep 3;29(9):117380. doi: 10.1016/j.isci.2026.117380 (PMC13571663; doi:10.1016/j.isci.2026.117380)
Supplement: Supplementary file 1 [file mmc1.pdf]

**iScience, Volume *29***

## Supplemental information

# *N*-propargylputrescine as a minimal-tag probe for imaging putrescine dynamics in cellular homeostasis and stress

**Aleksandra Owczarek, Aleksandra Duchnowska, Zuzanna Sas, Maciej Zakrzewski, Anita Florkowska, Martyna Nalepa, Aleksandra Skweres, Filip Suchożebski, Alicja Mąka, Jakub Szymanowski, Piotr Michaluk, Katarzyna Winiarska, Maciej Cieśla, Anna Marusiak, Remigiusz A. Serwa, and Michał Węgrzynowicz**

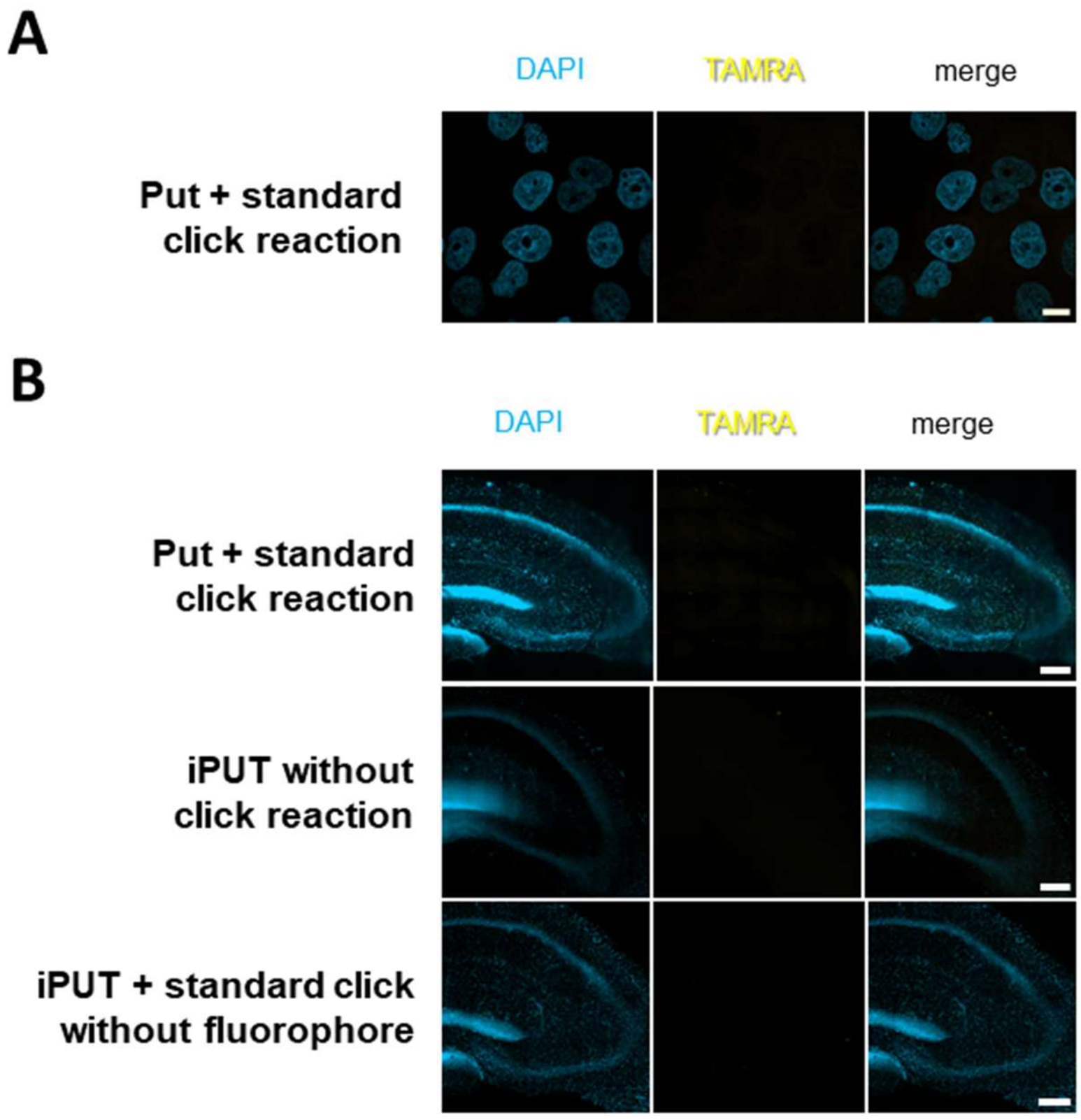


**Figure S1. Negative controls for iPUT experiments.**

(**A**) Negative control for MCF-7 cells. Representative confocal images of cells incubated with native Put instead of iPUT and clicked with TAMRA-5-azide (yellow). Nuclei are labelled with DAPI (cyan). Scale bar: 50 µm.

(**B**) Negative controls for acute mouse hippocampal slices. Top panel: representative confocal images of slices incubated with native Put instead of iPUT and clicked with TAMRA-5-azide (yellow). Nuclei are labelled with DAPI (cyan). Scale bar: 500µm. Middle panel: representative confocal images of slices incubated with iPUT without click reaction (yellow). Nuclei are labelled with DAPI (cyan). Scale bar: 500µm. Bottom panel: representative confocal images of slices incubated with iPUT and clicked in the absence of fluorophore (yellow). Nuclei are labelled with DAPI (cyan). Scale bar: 500µm.

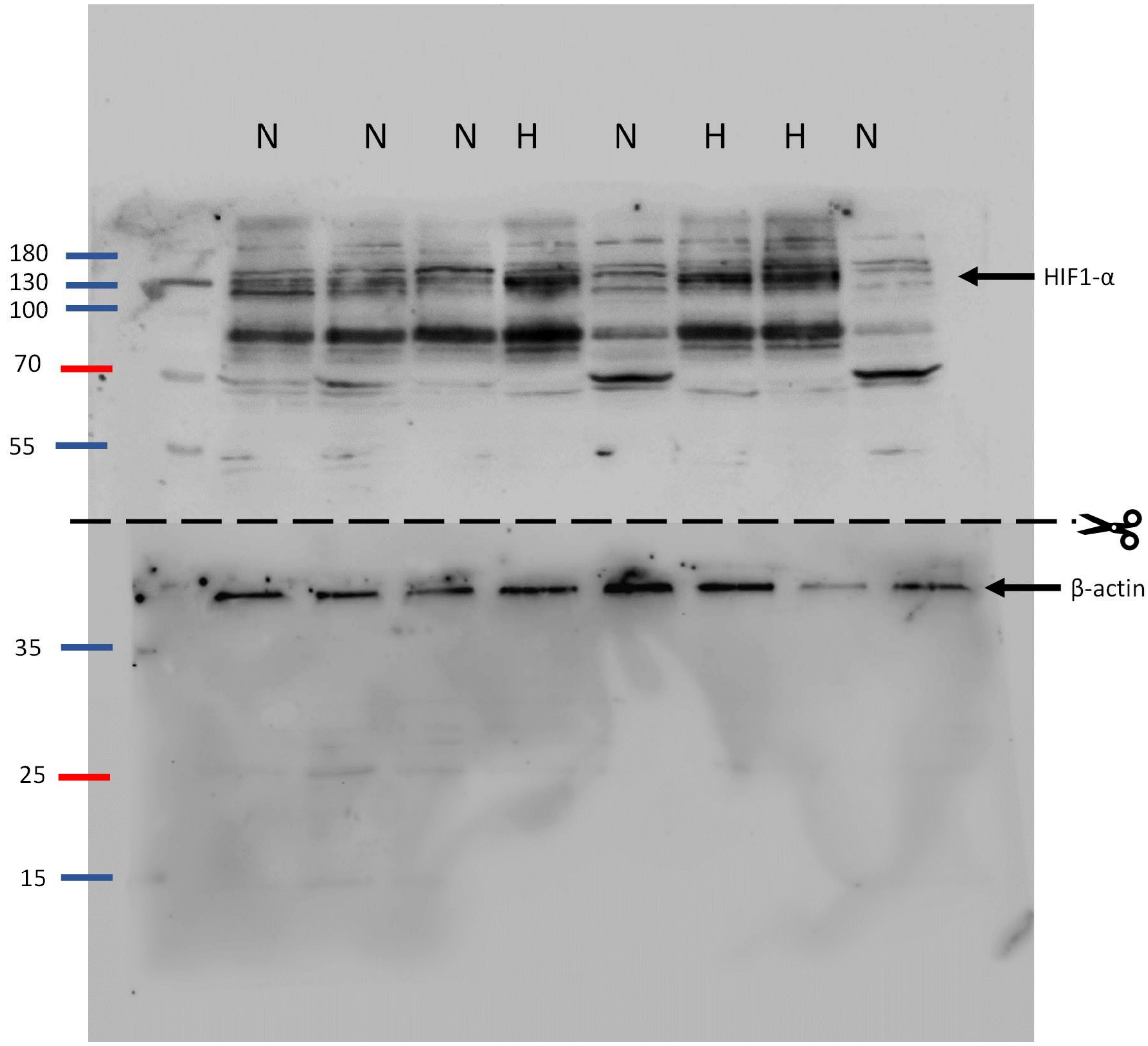


**Figure S2.** Unprocessed image of HIF-1α and β-actin (loading control) western blots in extracts from acute hippocampal slices incubated for 30 min in the absence or presence of $CoCl_2$. After blocking, the membrane was cut into two parts: the upper part was probed for HIF-1α and the lower part for β-actin. N – control, H – mimetic hypoxia (1 mM $CoCl_2$).
